# Existence of common fixed point and best proximity point for generalized nonexpansive type maps in convex metric space

**DOI:** 10.1186/s40064-016-3399-3

**Published:** 2016-11-09

**Authors:** Savita Rathee, Kusum Dhingra, Anil Kumar

**Affiliations:** 1 Department of Mathematics, Maharshi Dayanand University, Rohtak, Haryana 124001 India; 2 Department of Mathematics, Government College Bahu, Jhajjar, Haryana 124142 India

**Keywords:** Common property (E.A.), Common fixed point, Best proximity point, Compatible maps, q-affine, pq-affine, 46T99, 47H10, 54H25

## Abstract

Here, we extend the notion of (E.A.) property in a convex metric space defined by Kumar and Rathee (Fixed Point Theory Appl 1–14, 2014) by introducing a new class of self-maps which satisfies the common property (E.A.) in the context of convex metric space and ensure the existence of common fixed point for this newly introduced class of self-maps. Also, we guarantee the existence of common best proximity points for this class of maps satisfying generalized non-expansive type condition. We furnish an example in support of the proved results.

## Introduction and preliminaries

In 2002, Aamri and El Moutawakil (2002) obtained the notion of (E.A.) property for a single pair of self-maps. In the recent past, Liu et al. (2005) introduced common property (E.A.) and extend the concept of (E.A.) property defined by Aamri and El Moutawakil (2002) to two pairs of self-maps.

### **Definition 1**

(Liu et al. 2005) Two pairs (*A*, *S*) and (*B*, *T*) of self-maps of a metric space (*X*, *d*) are said to satisfy the common property (E.A.) if there exist two sequences $\{x_n\}$ and $\{y_{n}\}$ in *X* such that

$$\begin{aligned} \lim _{n\rightarrow \infty }Ax_n =\lim _{n\rightarrow \infty }Sx_n=\lim _{n\rightarrow \infty }By_n =\lim _{n\rightarrow \infty }Ty_n=t \quad {\text {for some}}\;t\in X. \end{aligned}$$

In 1970, Takahashi (1970) introduced the notion of convexity into metric space and proved several fixed point theorems for nonexpansive mappings in the context of convex metric space. Then after, Beg and Azam (1987), Fu and Huang (1991), Ciric (1993), and many others have obtained fixed point theorems in convex metric spaces. Very recently, Kumar and Rathee (2014) defined the concept of (E.A.) property in the setup of convex metric space and ensure the existence of common fixed point for a pair of maps satisfying this property by omitting the assumption that the range of one map is contained in other.

In the present work, we define the concept of common property (E.A.) in the context of convex metric space and extend the results of Kumar and Rathee (2014) to four self-maps by utilizing this newly introduced concept. Further, we ensure the existence of common best proximity point for generalized non-expansive type maps.

Before going to the main work, we recall some standard notations, known definitions and results which is required in the sequel. Throughout this paper, ${\mathbb {N}}$ and ${\mathbb {R}}$ denote the set of natural numbers and the set of real numbers, respectively.

### **Definition 2**

(Takahashi 1970) Let (*X*, *d*) be a metric space. A continuous mapping $W: X \times X \times [0,1] \rightarrow X$ is called a convex structure on X if, for all $x,y \in X$ and $\lambda \in [0,1]$, we have

1 $$\begin{aligned} d(u, W(x,y,\lambda ))\le \lambda d(u,x) + (1-\lambda )d(u,y) \end{aligned}$$

for all $$u\in X.$$

A metric space (*X*, *d*) equipped with a convex structure is called a convex metric space.

### **Definition 3**

A subset *M* of a convex metric space (*X*, *d*) is called a convex set (Takahashi 1970) if $W(x,y,\lambda ) \in M$ for all $x,y\in M$ and $\lambda \in [0,1]$. The set *M* is said to be *q*-starshaped (Guay et al. 1982) if there exists $q\in M$ such that $W(x,q,\lambda ) \in M$ for all $x\in M$ and $\lambda \in [0,1]$.

Clearly, each convex set *M* is starshaped with respect to any $q\in M$ but the converse assertion is not true. Thus, the class of starshaped set properly contains the class of convex set.

### **Definition 4**

(Guay et al. 1982) A convex metric space (*X*, *d*) is said to satisfy the Property (I), if for all $x, y, z \in X$ and $\lambda \in [0,1]$,

2 $$\begin{aligned} d(W(x,z,\lambda ), W(y,z,\lambda ))\le \lambda d(x,y). \end{aligned}$$

A normed linear space *X* and each of its convex subset are simple examples of convex metric spaces with *W* given by $W(x,y, \lambda ) = \lambda x + (1-\lambda )y$ for all $x,y\in X$ and $0\le \lambda \le 1$. Also, Property (I) is always satisfied in a normed linear space. There are many convex metric spaces which are not normed linear space, for details (see Guay et al. 1982; Takahashi 1970).

### **Definition 5**

Let (*X*, *d*) be a convex metric space and *M* be a subset of *X*. A mapping $I: M \rightarrow X$ is said to be

1. affine (Al-Thagafi and Shahzad 2006; Huang and Li 1996), if *M* is convex and $I(W(x,y,\lambda ))= W(Ix,Iy,\lambda )$ for all $x,y\in M$ and $\lambda \in [0,1]$.
2. *q*-affine (Al-Thagafi and Shahzad 2006; Kumar and Rathee 2014), if *M* is *q*-starshaped and $I(W(x,q,\lambda ))= W(Ix,q,\lambda )$ for all $x\in M$ and $\lambda \in [0,1]$.

### **Definition 6**

Let (*X*, *d*) be a metric space, M a nonempty subset of *X* and let *I* and *T* be self-maps of *M*. A point $x \in M$ is a coincidence point (common fixed point) of *I* and *T* if $Ix = Tx (Ix=Tx=x)$. The pair $\{I,T\}$ is called

1. commuting if $ITx=TIx$ for all $x \in M$.
2. compatible (Jungck 1986) if $\lim _{n\rightarrow \infty }d(ITx_m,TIx_m)=0,$ whenever $\{x_n\}$ is a sequence in *X* such that $\lim _{n\rightarrow \infty }Ix_n =\lim _{n\rightarrow \infty }Tx_n=t\in X$.

For more details about these classes, one can refer to (see Agarwal et al. 2014). In 1998, Pant (1998) defined the concept of reciprocal continuity as follows.

### **Definition 7**

(Pant 1998) Let (*X*, *d*) be a metric space and $I,T: X\rightarrow X$. Then the pair (*I*, *T*) is said to be reciprocally continuous if

$$\begin{aligned} \lim _{n\rightarrow \infty }ITx_n=It\quad \text {and}\quad \lim _{n\rightarrow \infty }TIx_n=Tt, \end{aligned}$$

whenever $\{x_n\}$ is a sequence in *X* such that $\lim _{n\rightarrow \infty }Ix_n =\lim _{n\rightarrow \infty }Tx_n=t\in X$.

It is easy to see that if *I* and *T* are continuous, then the pair (*I*, *T*) is reciprocally continuous but the converse is not true in general (see Imdad et al. 2011, Example 2.3). Moreover, in the setting of common fixed point theorems for compatible pairs of self-mappings satisfying some contractive conditions, continuity of one of the mappings implies their reciprocal continuity.

### **Definition 8**

(Bouhadjera and Godet-Thobie 2009) Let *I* and *T* be two self-maps of a metric space (*X*, *d*). Then the pair (*I*, *T*) is said to be subcompatible if there exists a sequence $\{x_n\}$ such that

$$\begin{aligned} \lim _{n\rightarrow \infty }Ix_n =\lim _{n\rightarrow \infty }Tx_n=t\in X, \quad {\text {for some}}\;t \in X \;\; \text {and}\;\; \lim _{n\rightarrow \infty }d(ITx_n,TIx_n)=0. \end{aligned}$$

Obviously, compatible maps which satisfy (E.A.) property are subcompatible but the converse statement does not hold in general (see Rouzkard et al. 2012, Example 2.5)

### **Definition 9**

(Kumar and Rathee 2014) Let *M* be a *q*-starshaped subset of a convex metric space (*X*, *d*) and let $I,T:M\rightarrow M$ with $q\in F(I)$. The pair (*I*, *T*) is said to satisfy (E.A.) property with respect to *q* if there exists a sequence $\{x_n\}$ in *M* such that for all $\lambda \in [0,1]$

3 $$\begin{aligned} \lim _{n\rightarrow \infty }Ix_n = \lim _{n\rightarrow \infty }T_{\lambda }x_n = t\in M, \end{aligned}$$

where $$T_{\lambda }x = W(Tx,q,\lambda )$$.

Obviously, if the pair (*I*, *T*) satisfy (*E*.*A*.) property with respect to *q*, then *I* and *T* satisfy (*E*.*A*.) property but converse assertion is not necessarily true (see Kumar and Rathee 2014, Example 12).

## Main results

We start to this section with following definition.

### **Definition 10**

Let *M* be a *q*-starshaped subset of a convex metric space (*X*, *d*) and let *A*, *B*, *S* and $T:M\rightarrow M.$ Two pairs (*A*, *S*) and (*B*, *T*) are said to satisfy common property (*E*.*A*.) with respect to *q* if there exist two sequences $\{x_n\}$ and $\{y_n\}$ in *M* such that for all $\lambda \in [0,1]$

4 $$\begin{aligned} \lim _{n\rightarrow \infty }Ax_n = \lim _{n\rightarrow \infty }S_{\lambda }x_n =\lim _{n\rightarrow \infty }By_n = \lim _{n\rightarrow \infty }T_{\lambda }y_n = t\in M, \end{aligned}$$

where $$S_{\lambda }x = W(Sx,q,\lambda )$$ and $$T_{\lambda }y = W(Ty, q, \lambda )$$

### *Remark 11*

In Definition 10, if $A=B$ and $S=T$, then Definition 9 can be obtained as a particular case of Definition 10. Therefore the common property (E.A.) defined here extends the notion of (E.A.) property in convex metric space defined by Kumar and Rathee (2014).

The following Lemma is particular case of the Theorem 4.1 of Chauhan and Pant (2014).

### **Lemma 12**

*Let* *A*, *B*, *S* *and* *T* *be self-maps of a metric space* (*X*, *d*). *If the pairs* (*A*, *S*) *and* (*B*, *T*) *are subcompatible, reciprocally continuous and satisfy*

5 $$\begin{aligned} d(Sx,Ty)\le \lambda \max \{d(Ax,By), d(Ax,Sx), d(By,Ty),d(Ax,Ty),d(By,Sx)\} \end{aligned}$$

*for some* $\lambda \in (0,1)$ *and all* $x,y\in X$. *Then* *S* *and* *T* *have a unique common fixed point in* *X*.

Now, we start with the following theorem.

### **Theorem 13**

*Let* *M* *be a nonempty* *q*-*starshaped subset of a convex metric space* (*X*, *d*) *with Property (I) and let* *A*, *B*, *S* *and* *T* *be continuous self-maps on* *M* *such that the pair* (*A*, *S*) *and* (*B*, *T*) *satisfying common property* (*E*.*A*.) *w.r.t. q. Assume that* *A* *and* *B* *are q-affine, M is compact. If* *A*, *B*, *S* *and* *T* *are compatible and satisfy the inequality*

6 $$\begin{aligned} d(Sx, Ty) \le \max \left\{ d(Ax, By), dist(Ax, [sx,q]),dist(By, [Ty,q]), dist(Ax, [Ty, q]), dist(By, [Sx,q])\right\} \end{aligned}$$

*for all* $x, y \in M$, *then* $M \bigcap F(A) \bigcap F(B) \bigcap F(S) \bigcap F(T)\neq\phi$.

### *Proof*

For each $n \in N$, we define $T_{n} : M \rightarrow M$ and $S_n: M \rightarrow M$ by

7 $$\begin{aligned} T_{n}(y) = W(Ty, q, \lambda _{n}) \;and \; S_{n}(x) = W(Sx, q, \lambda _{n}) \end{aligned}$$

for all $x \in M$, where $\lambda _{n}$ is a sequence in (0, 1) such that $\lambda _{n} \rightarrow 1.$ Now we have to prove that for each $n \in N$, the pair $(S_{n}, A)$ and $(T_{n}, B)$ are subcompatible. Since *A*, *B*, *S* and *T* are satisfying the common property (*E*.*A*.) w.r.t. *q*, there exist two sequences $\left\{ x_{m}\right\}$ and $\left\{ y_{m}\right\}$ in *M* such that for all $\lambda \in [0,1]$

8 $$\begin{aligned} \lim _{m \rightarrow \infty }Ax_{m} =\lim _{m \rightarrow \infty }S_{\lambda }(x_{m})= \lim _{m \rightarrow \infty }By_{m} = \lim _{m \rightarrow \infty } T_{\lambda }y_{m} =t \end{aligned}$$

for $$t \in M,$$ where

$$\begin{aligned} \lim _{m \rightarrow \infty }T_{\lambda }y_{m}&= \lim _{m \rightarrow \infty }W(Ty_m, q, \lambda )\\ {\hbox{and}} \lim _{m \rightarrow \infty }S_{\lambda }x_m&= \lim _{m \rightarrow \infty }W(Sx_m, q, \lambda ) .\end{aligned}$$

Since $\lambda _{n} \in (0,1)$, by using Eqs. (7) and (8) for each $n \in N$, we have

$$\begin{aligned} \lim _{m \rightarrow \infty }T_{n}y_{m}&= \lim _{m \rightarrow \infty }W(Ty_{m}, q, \lambda _{n})\\&= \lim _{m \rightarrow \infty }T_{\lambda _{n}}(y_{m})\\&= t \in M. \end{aligned}$$

Thus we have

9 $$\begin{aligned} \lim _{m \rightarrow \infty }By_{m} = \lim _{m \rightarrow \infty }T_{n}y_{m} = t \in M. \end{aligned}$$

Similarly

$$\begin{aligned} \lim _{m \rightarrow \infty }S_{n}x_{m}&= \lim _{m \rightarrow \infty }W(Sx_{m}, q, \lambda _{n})\\&= \lim _{m \rightarrow \infty }S_{\lambda _{n}}x_{m}\\&= t \in M. \end{aligned}$$

and so

10 $$\begin{aligned} \lim _{m \rightarrow \infty }Ax_{m} = \lim _{m \rightarrow \infty }S_{n}x_{m} = t \in M. \end{aligned}$$

Hence in light of Eqs. (9) and (10), we obtain

11 $$\begin{aligned} \lim _{m \rightarrow \infty }Ax_{m} = \lim _{m \rightarrow \infty }S_{n}x_{m} = \lim _{m \rightarrow \infty }By_{m} = \lim _{m \rightarrow \infty }T_{n}y_{m} = t \in M. \end{aligned}$$

Taking in to account that *A* and *B* are *q*-affine and by using Property (*I*), we have

12 $$\begin{aligned} d(S_{n}Ax_{m}, AS_{n}x_{m})&= d(W(SAx_{m},q, \lambda _{n}), A(W(Sx_{m},q, \lambda _{n})))\nonumber \\&= d(W(SAx_{m},q, \lambda _{n}),W(ASx_{m},q, \lambda _{n}))\nonumber \\&\le \lambda _{n}d(SAx_{m}, ASx_{m}). \end{aligned}$$

Similarly, we can show that

13 $$\begin{aligned} d(T_{n}By_{m}, BT_{n}y_{m}) \le \lambda _{n}d(TBy_{m}, BTy_{m}). \end{aligned}$$

As (*A*, *S*) and (*B*, *T*) satisfy (*E*.*A*.) property w.r.t.*q* then these pairs also satisfy (*E*.*A*.) property and hence by using the compatibility of *A*, *S*, *B*,and *T* we get

$$\begin{aligned} \lim _{m \rightarrow \infty }d(BTy_{m}, TBy_{m})&= 0\\ {\hbox{and}} \lim _{m \rightarrow \infty } d(ASx_{m}, SAx_{m})&= 0. \end{aligned}$$

Taking limit $m \rightarrow \infty$ in Eqs. (12) and (13), we obtain

14 $$\begin{aligned} \lim _{m \rightarrow \infty }d(BT_{n}y_{m}, T_{n}By_{m}) = \lim _{m \rightarrow \infty } d(AS_{n}x_{m}, S_{n}Ax_{m}) = 0. \end{aligned}$$

Taken into account Eqs. (11) and (14), it follows that $(T_{n}, B)$ and $(S_{n}, A)$ are subcompatible for each $n \in N$. Since *A*, *B*, *S* and *T* are continuous for each $n \in N$, the pair $(S_{n}, A)$ and $(T_{n},B)$ are reciprocally continuous.

By using equation (6) and Property (I), we get that

15 $$\begin{aligned} d(S_{n}x, T_{n}y)&= d(W(Sx, q, \lambda _{n}),W(Ty, q, \lambda _{n})) \nonumber \\&\le \lambda _{n}d(Sx, Ty)\nonumber \\&\le \lambda _{n}\max \left\{ d(Ax, By), dist(Ax, [Sx,q]),dist(By,[Ty,q]),dist(Ax,[Ty,q]),dist(By,[Sx,q])\right\} \nonumber \\&\le \lambda _{n}\max \left\{ d(Ax, By), d(Ax, S_{n}x), d(By, T_{n}y), d(Ax, T_{n}y), d(By, S_{n}x)\right\} \end{aligned}$$

for each $x, y \in M$ and $\lambda _{n} \in (0, 1)$. By Lemma 12, for each $n \in N$, there exists $x_{n} \in M$ such that

$$\begin{aligned} Ax_{n} = S_{n}x_{n}=Bx_{n}= T_{n}x_{n}=x_{n}. \end{aligned}$$

Now by taking the compactness of *M*, we know continuous image of compact set is compact so *T*(*M*) and *S*(*M*) are compact and every compact set is sequentially compact. Therefore there exist subsequences $\left\{ Tx_{m}\right\}$ of $\left\{ Tx_{n}\right\}$ and $\left\{ Sx_{m}\right\}$ of $\left\{ Sx_{n}\right\}$ such that $\lim _{m \rightarrow \infty }Tx_{m} =z$ and $\lim _{m \rightarrow \infty }Sx_{m} =y.$

Now, we have to prove that $y =z.$

On the contrary suppose that $y \ne z$, then we have

$$\begin{aligned}&x_{m} = T_{m}x_{m} = W(Tx_{m},q,\lambda _{m}) \rightarrow z\hbox { as }m \rightarrow \infty \\&\hbox {and }x_{m} = S_{m}x_{m} = W(Sx_{m},q,\lambda _{m}) \rightarrow y \hbox { as }m \rightarrow \infty. \end{aligned}$$

This implies that the sequence $\left\{ x_{m}\right\}$ converges to two points which is contradiction. Hence $y=z$.

Since $x_{m} \rightarrow z$ as $m \rightarrow \infty$ and the mappings *A*, *B*, *S* and *T* are continuous, it follows

$$\begin{aligned} Az = Tz = Sz = Bz = z. \end{aligned}$$

So, z is common fixed point of *A*, *B*, *S* and *T.*

This implies that $M \bigcap F(A) \bigcap F(B) \bigcap F(S) \bigcap F(T) \ne \phi$. $\square$

### **Corollary 14**

*Let* *M* *be a nonempty* *q*-*starshaped subset of a convex metric space* (*X*, *d*) *with Property (I) and let* *A*, *B*, *S* *and* *T* *be continuous self-maps on* *M* *such that the pair* (*A*, *S*) *and* (*B*, *T*) *satisfying common property* (*E*.*A*.) *w.r.t. q. Assume that* *A* *and* *B* *are q-affine, M is compact. If* *A*, *B*, *S* *and* *T* *are compatible and satisfy the inequality*

16 $$\begin{aligned} d(Sx, Ty) \le \max \left\{ d(Ax, By), dist(Ax, [sx,q]),dist(By, [Ty,q]), \frac{1}{2}[dist(Ax, [Ty, q])+ dist(By, [Sx,q])]\right\} \end{aligned}$$

*for all* $x, y \in M$, *then* $M \bigcap F(A) \bigcap F(B) \bigcap F(S) \bigcap F(T) \ne \phi.$

### **Corollary 15**

*Let* *M* *be a nonempty* *q*-*starshaped subset of a convex metric space* (*X*, *d*) *with* $Property \;(I)$ *and let* *A*, *B*, *S* *and* *T* *be* *continuous self-maps on* *M* *such that the pair* (*A*, *S*) *and* (*B*, *T*) *satisfying common property* (*E*.*A*.) *w.r.t. q. Assume that* *A* *and* *B* *are q-affine, M is compact. If* *A*, *B*, *S* *and* *T* *are R-subweakly commuting and satisfy the inequality*

17 $$\begin{aligned} d(Sx, Ty) \le \max \left\{ d(Ax, By), dist(Ax, [sx,q]),dist(By, [Ty,q]), dist(Ax, [Ty, q]), dist(By, [Sx,q])\right\} \end{aligned}$$

*for all* $x, y \in M,$ *then* $M \bigcap F(A) \bigcap F(B) \bigcap F(S) \bigcap F(T) \ne \phi.$

Now we present an example in support of our theorem.

### *Example 16*

Let $X=R$ endowed with usual metric and let $M= \left[ -1,\frac{2}{3}\right]$. Define *A*, *B*, *S* and $T:M \rightarrow M$ by:

$$\begin{aligned} A(x)&= {\left\{ \begin{array}{ll} \frac{1}{3} &{} \quad {\text {if}}\;-1\le x\le \frac{1}{3} \\ \frac{5}{3}-4x &{} \quad {\text {if}}\;\frac{1}{3}\le x\le \frac{2}{3} \end{array}\right. } \quad \text {and}\quad S(x) = {\left\{ \begin{array}{ll} \frac{1}{3} &{} {\text {if}}\;-1\le x \le \frac{1}{3} \\ \frac{x}{2}+\frac{1}{6} &{}\quad {\text {if}}\;\frac{1}{3}\le x\le \frac{2}{3} \end{array}\right. }\\ B(x)&= {\left\{ \begin{array}{ll} \frac{1}{3} &{}\quad {\text {if}}\;-1\le x\le \frac{1}{3} \\ 1-2x &{}\quad {\text {if}}\;\frac{1}{3}\le x\le \frac{2}{3} \end{array}\right. } \quad \text {and}\quad T(x) = {\left\{ \begin{array}{ll} \frac{1}{3} &{}\quad {\text {if}}\;-1\le x \le \frac{1}{3} \\ \frac{x}{4}+\frac{1}{4} & \quad {\text {if}}\;\frac{1}{3}\le x\le \frac{2}{3}. \end{array}\right.} \end{aligned}$$

Then (*X*, *d*) is a convex metric space with the convex structure $W(x, y, \lambda ) = (\lambda )x + (1-\lambda )y$.

We have to check the following:

(i): *A* and *B* are *q*-affine with $q =\frac{1}{3}$
(ii): The pair (*A*, *S*) and (*B*, *T*) satisfying common property (*E*.*A*.) w.r.t. $q = \frac{1}{3}$.
(iii): *A*, *B*, *S* and *T* are compatible.

### *Proof*

(i) If $$x \in \left[ -1, \frac{1}{3}\right]$$, then $$W\left( x, \frac{1}{3}, \lambda \right) = (\lambda )x +(1 - \lambda )\frac{1}{3} \in \left[ -1, \frac{1}{3}\right]$$.

That implies $$A\left( W\left( x, \frac{1}{3}, \lambda \right) \right) = W\left( Ax, \frac{1}{3}, \lambda \right) .$$

Again, if $x \in \left[ \frac{1}{3}, \frac{2}{3}\right]$, then $W\left( x, \frac{1}{3}, \lambda \right) = (\lambda )x +(1 - \lambda )\frac{1}{3} \in \left[ \frac{1}{3}, \frac{2}{3}\right]$, so we get

$$\begin{aligned} A\left( W\left( x, \frac{1}{3}, \lambda \right) \right)&= \frac{5}{3} - 4\left( W\left( x, \frac{1}{3}, \lambda \right) \right) \\&= \frac{5}{3} -4\lambda x-4(1-\lambda )\frac{1}{3}\\&= \frac{1}{3} -4\lambda x + \frac{4}{3}\lambda \\&= \frac{1}{3} + 4\lambda \left( \frac{1}{3}-x\right) \\ \end{aligned}$$

and

$$\begin{aligned} W\left( Ax, \frac{1}{3}, \lambda \right)&= W\left( \frac{5}{3} - 4x, \frac{1}{3}, \lambda \right) \\&= \lambda \left( \frac{5}{3} -4x\right) +(1-\lambda )\frac{1}{3}\\&= \frac{5}{3}\lambda -4\lambda x + \frac{1}{3}-\lambda \left( \frac{1}{3}\right) \\&= \frac{1}{3} + 4\lambda \left( \frac{1}{3}-x\right) . \end{aligned}$$

Thus, $A\left( W\left( x,\frac{1}{3},\lambda \right) \right) = W\left( Ax, \frac{1}{3},\lambda \right)$ for all $x\in M$ and hence *A* is q-affine with $q=\frac{1}{3}.$

Now we shall prove that *B* is *q*-affine with $q = \frac{1}{3}.$

For this, if $x \in \left[ -1,\frac{1}{3}\right]$, then $B\left( W\left( x, \frac{1}{3}, \lambda \right) \right) = W\left( Bx, \frac{1}{3}, \lambda \right) .$ and if $x \in \left[ \frac{1}{3}, \frac{2}{3}\right]$, then $W\left( x, \frac{1}{3}, \lambda \right) = (\lambda )x +(1 - \lambda )\frac{1}{3} \in \left[ \frac{1}{3}, \frac{2}{3}\right]$. Therefore, we have

$$\begin{aligned} B\left( W\left( x, \frac{1}{3}, \lambda \right) \right)&= 1 - 2\left( W\left( x, \frac{1}{3}, \lambda \right) \right) \\&= 1-2\lambda x-2(1-\lambda )\frac{1}{3}\\&= \frac{1}{3} + 2\lambda \left( \frac{1}{3}-x\right) \end{aligned}$$

and

$$\begin{aligned} W\left( Bx, \frac{1}{3}, \lambda \right)&= \lambda (1 - 2x)+(1-\lambda ) \frac{1}{3}\\&= \lambda -2\lambda x+\frac{1}{3}-\frac{1}{3}\lambda \\&= \frac{1}{3} + 2\lambda \left( \frac{1}{3}-x\right) . \end{aligned}$$

So, $B\left( W\left( x, \frac{1}{3}, \lambda \right) \right) = W\left( Bx, \frac{1}{3}, \lambda \right)$ for each $x\in M$. This implies that *B* is *q*-affine with $q= \frac{1}{3}$. $\square$

### *Proof*

(ii) Clearly $$A\left(\frac{1}{3}\right) =B\left(\frac{1}{3}\right) =\frac{1}{3}.$$

Consider $x_{n} = \frac{1}{3} -\frac{1}{n+2}, n \ge 1$ and $y_{n} = \frac{1}{3} -\frac{1}{3n}, n\ge 1$ then for each $n, x_{n}$ and $y_{n} \in [0,\frac{1}{3}]$ and for each $\lambda \in [0,1]$, we have

$$\begin{aligned}&\limsup _{n \rightarrow \infty }S_{\lambda }x_{n} = W \left( \frac{1}{3}, \frac{1}{3}, \lambda \right) = \frac{1}{3} = \lim _{n \rightarrow \infty }Ax_{n}\\&\hbox { and }\limsup _{n \rightarrow \infty }T_{\lambda }y_{n} = W \left( \frac{1}{3}, \frac{1}{3}, \lambda \right) = \frac{1}{3} = \lim _{n \rightarrow \infty }By_{n}. \end{aligned}$$

This implies that the pair (*A*, *S*) and (*B*, *T*) satisfying common property (E.A.) with respect to $q = \frac{1}{3}.$ $\square$

### *Proof*

(iii) Here, we shall prove that the pairs (*A*, *S*) and (*B*, *T*) are compatible.

If $\{x_{n}\}$ and $\{y_{n}\}$ are two sequences in *M* such that

$$\begin{aligned}&\lim _{n \rightarrow \infty }Ax_{n} = \lim _{n \rightarrow \infty }Sx_{n} = t \quad {\hbox {for some}} \;t {\hbox { and }}\\&\lim _{n \rightarrow \infty }By_{n} = \lim _{n \rightarrow \infty }Ty_{n} = k \quad {\hbox {for some}} \; k. \end{aligned}$$

Then *t* and *k* lies in the closure of *A*(*M*), *S*(*M*) and *B*(*M*), *T*(*M*) respectively, where

$$\begin{aligned}&Cl(A(M)) = \left[ -1, \frac{1}{3}\right] \quad {\text {and}}\quad Cl(S(M)) = \left[ \frac{1}{3}, \frac{1}{2}\right] \\&Cl(B(M)) = \left[ \frac{-1}{3}, \frac{1}{3}\right] \quad {\text {and}}\quad Cl(T(M)) = \left[ \frac{1}{3}, \frac{5}{12}\right]. \end{aligned}$$

So $$t=k=\frac{1}{3}.$$

Therefore, by using the continuity of *A*, *B*, *S* and *T*, we have

$$\begin{aligned}&\lim _{n \rightarrow \infty }ASx_{n} = A\lim _{n \rightarrow \infty }Sx_{n} = A\left( \frac{1}{3}\right) = \frac{1}{3}\\&\lim _{n \rightarrow \infty }SAx_{n} = S\lim _{n \rightarrow \infty }Ax_{n} = S\left( \frac{1}{3}\right) = \frac{1}{3}. \end{aligned}$$

This implies that the pair (*A*, *S*) is compatible. Similarly we can prove that the pair (*B*, *T*) is compatible. $\square$

Finally we have to prove the inequality (6). There are two possibilities: (i) $x = y$ and (ii) $x \ne y.$

(i) If $$x =y$$, then
  Subcase (i): if $x= y \in \left[ -1, \frac{1}{3}\right]$, then $d(Sx, Tx) = 0.$ So the inequality holds trivially.
  Subcase (ii): if $x =y \in \left[ \frac{1}{3}, \frac{2}{3}\right]$, then
  $$\begin{aligned} d(Sx, Tx)&= \left| \frac{x}{2} + \frac{1}{6}-\frac{x+1}{4}\right| \\&= \left| \frac{6x+2-3x-3}{12}\right| \\&= \left| \frac{3x-1}{12}\right| \\&= \frac{1}{4}\left| x-\frac{1}{3}\right| \\ d(Ax,Bx)&= \left| \frac{5}{3} -4x-1+2x \right| \\&= \left| \frac{1}{3} -2x\right| \\&= 2\left| x-\frac{1}{3}\right|. \end{aligned}$$
  That implies $$d(Sx,Tx) \le d(Ax,Bx).$$
(ii) If $x \ne y$, then
  Subcase (i): if $x \ne y \in [0, \frac{1}{3}]$, then $d(Sx, Ty) = 0$. Inequality trivially holds.
  Subcase (ii): if $x \ne y \in \left[ \frac{1}{3}, \frac{2}{3}\right]$, then
  $$\begin{aligned} d(Sx, Ty)&= \left| \frac{x}{2} + \frac{1}{6}-\frac{y+1}{4}\right| \\&= \left| \frac{6x+2-3y-3}{12}\right| \\&= \left| \frac{6x-3y-1}{12}\right| \\&= \frac{1}{2}\left| x-\frac{y}{2}- \frac{1}{6}\right| \\ d(Ax,By)&= \left| \frac{5}{3} -4x-1+2y \right| \\&= \left| \frac{2}{3} -4x +2y\right| \\&= 4\left| x-\frac{y}{2}-\frac{1}{6}\right|. \end{aligned}$$
  This implies $$d(Sx,Ty) \le d(Ax, By).$$
  Subcase (iii): if $x \in \left[ -1,\frac{1}{3}\right]$ and $y \in \left[ \frac{1}{3}, \frac{2}{3}\right]$, then
  $$\begin{aligned} d(Sx, Ty)&= \left| \frac{1}{3} -\frac{y+1}{4}\right| \\&= \left| \frac{4-3y-3}{12}\right| \\&= \left| \frac{1-3y}{12}\right| \\&= \frac{1}{4}\left| \frac{1}{3}-y\right| \\ d(Ax,By)&= \left| \frac{1}{3} -1+2y \right| \\&= \left| 2y-\frac{2}{3} \right| \\&= 2\left| \frac{1}{3}-y\right|. \end{aligned}$$
  Therefore, we get $d(Sx,Ty) \le d(Ax, By).$
  Subcase (iv): if $x \in \left[ \frac{1}{3}, \frac{2}{3}\right]$ and $y \in \left[ -1, \frac{1}{3}\right]$, then
  $$\begin{aligned} d(Sx, Ty)&= \left| \frac{x}{2} -\frac{1}{6}\right| \\&= \frac{1}{2}\left| x-\frac{1}{3}\right| \\ d(Ax,By)&= \left| \frac{5}{3} -4x-\frac{1}{3} \right| \\&= 4\left| \frac{1}{3}-x\right|. \end{aligned}$$
  So, we have $d(Sx,Ty) \le d(Ax, By).$

Thus, for each $x,y \in M$, the mappings *A*, *B*, *S* and *T* satisfying the inequality (6). Also *M* is compact and *A*, *B*, *S* and *T* are continuous. Thus we conclude that *A*, *B*, *S* and *T* satisfying all the conditions of Theorem 13 and consequently

$$\begin{aligned} M \bigcap F(A) \bigcap F(B) \bigcap F(S) \bigcap F(T) \ne \phi. \end{aligned}$$

Here $\frac{1}{3} \in M$ is such a common fixed point of *A*, *B*, *S* and *T*.

### *Remark 17*

It is to be noted that, in Example 16, $S(M) \not \subset A(M)$ and $T(M)\not \subset B(M)$. Therefore all the existing common fixed point theorems which ensure the existence of common fixed point for the maps under the hypothesis that range of one set is contained in other are not applicable to Example 16 (see Chen and Li (2007), Rathee and Kumar (2014a, b), Shahzad (2001)).

## Application to invariant approximation

For a nonempty subset *M* of a metric space (*X*, *d*) and $p\in X$, an element $y\in M$ is called a best approximation to *p* if $d(p,y)=dist(p,M)$, where $dist(p,M) = \inf \{d(p,z): z\in M\}$. The set of all best approximations to *p* is denoted by $P_M(p)$.

As an application of Theorem 13, we present an invariant approximation theorems.

### **Theorem 18**

*Let* *A*, *B*, *S* *and* *T* *be self-maps of a convex metric space* (*X*, *d*) *with Property (I)*, $p\in F(S)\cap F(T)\cap F(A) \cap F(B)$, *and* *M* *be a subset of* *X* *such that* $S(\delta M\cap M)\subseteq M$ *and* $T(\delta M\cap M)\subseteq M$, *where* $\delta M$ *denotes the* *boundary of* *M*. *Suppose that* $P_M(p)$ *is nonempty*, *q* *-starshaped* *with* $A(P_M(p))\subset P_M(p)$ *and* $B(P_M(p))\subset P_M(p)$ *and* *also the maps* *A* *and* *B* *are q-affine and continuous on* $P_M(p)$. *If the pairs* (*A*, *S*) *and* (*B*, *T*) *are compatible, satisfy the common property (E.A.) w.r.t.* *q* *and also satisfy the inequality for all* $x,y\in P_M(p)\cup \{p\}$

18 $$\begin{aligned}&d(Tx,Sy)\le {\left\{ \begin{array}{ll} \ d(Ax,Bp) &{} {\text {if}}\;y = p \\ \max \{d(Ax, By), dist(Ax, [sx,q]),dist(By, [Ty,q]), \\ dist(Ax, [Ty, q]), dist(By, [Sx,q])\} &{} \text { if }y\in P_M(p),\\ \end{array}\right. }\nonumber \\&d(Sx, Tp)\le d(Tx,Sp). \end{aligned}$$

*Then* *A*, *B*, *S* *and* *T* *have a common fixed point in* $P_M(p)$, *provided that* $P_M(p)$ *is compact and the maps* *T* *and* *S* *are continuous on* $P_M(p)$.

### *Proof*

Let $x\in P_M(p)$. Then for all $\lambda \in (0,1)$, we have

$$\begin{aligned} d(p, W(x,p,\lambda ))\le \lambda d(p,x)+(1-\lambda )d(p,p)=\lambda d(p,x) < dist(p,M). \end{aligned}$$

Therefore $W(x,p,\lambda )\notin M$ for any $\lambda \in (0,1)$ and hence $x\in \delta M\cap M$. Thus, as $S(\delta M\cap M)\subseteq M$ and $T(\delta M\cap M)\subseteq M$, we have $Tx\in M$ and $Sx\in M$. Also, since $Ax\in P_M(p)$ and $p\in F(S)\cap F(T)\cap F(A) \cap F(B)$, by using Eq. (18), we get

$$\begin{aligned} d(Tx,p)=d(Tx,Sp)\le d(Ax,Bp)=d(Ax,p)=dist(p,M) \end{aligned}$$

and

$$\begin{aligned} d(Sx,p)= d(Sx,Tp)\le d(Tx,Sp)\le d(Ax,Bp)=d(Ax,p)=dist(p,M). \end{aligned}$$

Thus, $Tx\in P_M(p)$ and $Sx\in P_M(p)$. So *A*, *B*, *S* and *T* are self-maps on $P_M(p)$. In view of Theorem 13, we can say that *A*, *B*, *S* and *T* have a common fixed point in $P_M(p)$. $\square$

Define $$D= P_M(p)\cap C_M^{A,B}(p)$$, where $$C_M^{A,B}(p)=\{x\in M : Ax\in P_M(p)\quad \text {and}\quad Bx\in P_M(p)\}$$

### **Theorem 19**

*Let* *A*, *B*, *S* *and* *T* *be self-maps of a convex metric space* (*X*, *d*) *with Property (I)*, $p\in F(S)\cap F(T)\cap F(A) \cap F(B)$, *and* *M* *be a subset of* *X* *such that* $S(\delta M\cap M)\subseteq M$ *and* $T(\delta M\cap M)\subseteq M$, *where* $\delta M$ *denotes the boundary of* *M*. *Suppose that* *D* *is nonempty*, *q*-*starshaped with* $A(D)\subset D$ *and* $B(D)\subset D$ *and also the maps* *A* and *B* *are q-affine and nonexpansive on* *D*. *If the pairs* (*A*, *S*) *and* (*B*, *T*) *are compatible, satisfy the common property (E.A.) w.r.t.* *q* *and also satisfy the inequality for all* $x,y\in D\cup \{p\}$

19 $$\begin{aligned}&d(Tx,Sy)\le {\left\{ \begin{array}{ll} \ d(Ax,Bp) &{} {\text {if}}\;y = p \\ \max \{d(Ax, By), dist(Ax, [sx,q]),dist(By, [Ty,q]), \\ dist(Ax, [Ty, q]), dist(By, [Sx,q])\} &{} \text { if }y\in D,\\ \end{array}\right. }\nonumber \\&d(Sx, Tp)\le d(Tx,Sp). \end{aligned}$$

*Then* *A*, *B*, *S* *and* *T* *have a common fixed point in* $P_M(p)$, *provided that* *D* *is compact and the maps* *T* *and* *S* *are continuous on* *D*.

### *Proof*

Let $x\in D$. Then by following the steps as we have done in Theorem 18, we get that $Tx\in P_M(p)$ and $Sx\in P_M(p)$. Since the maps *A* and *B* are nonexpansive and $p\in F(S)\cap F(T)\cap F(A) \cap F(B)$, by using Eq. 19, we have

$$\begin{aligned} d(ATx,p)=d(ATx,Ap)\le d(Tx,p)= d(Tx,Sp)\le d(Ax,p)=dist (p,M) \end{aligned}$$

and

$$\begin{aligned} d(BTx,p)=d(BTx,Bp)\le d(Tx,p)= d(Tx,Sp)\le d(Ax,p)=dist (p,M). \end{aligned}$$

That imply *ATx* and $BTx \in P_M(p)$ and hence $Tx\in C_M^{A,B}(p)$. Similarly we can show that $Sx\in C_M^{A,B}(p)$. Thus we can say *A*, *B*, *S* and *T* are self-maps on *D* and so Theorem 13 guarantees the existence of $z\in P_M(p)$ such that *z* is a common fixed point of *A*, *B*, *S* and *T*. $\square$

## Best proximity point

First we discuss the concept of best proximity. Let $T:A \rightarrow B$ be a map where *A* and *B* are two nonempty subsets of a metric space (*X*, *d*) and let *A* and *B* are disjoint subsets of a metric space then the equation $Tx=x$ might have no solution. Therefore in case of nonself-maps we are not sure about the existence of fixed point. In such a case we try to minimize the distance *d*(*x*, *Tx*) and a point *x* for which *d*(*x*, *Tx*) is minimum is called a best proximity point. In the recent years there have been many interesting best proximity point theorems are proved, for example, see De la Sen et al. (2013), Eldred and Veeramani (2006), Prolla (1983), Reich (1978), and Sankar Raj (2011), Sehgal and Singh (1988). In the present section we prove a new best proximity theorem for four maps but before this we recall some definitions which are required in the sequel.

### **Definition 20**

Let (*X*, *d*) be a convex metric space and *A*, *B* be two nonempty subsets of *X*. A mapping $f:A \rightarrow B$ is called *pq*-affine if

(i): *A* is *p*-starshaped set and *B* is *q*-starshaped set;
(ii): $$f(W(x,p, \lambda )) = W(fx,q,\lambda )$$.

### **Definition 21**

Let *A* and *B* be two nonempty subsets of convex metric space (*X*, *d*). Let *A* be a *p*-starshaped set and *B* be a *q* starshaped set. Let *f*, *g*, *S*,  and *T* be four nonself-maps from *A* to *B*. Two pairs (*f*, *S*) and (*g*, *T*) are said to satisfy common property (*E*.*A*.) with respect to *q* if there exists two sequences $\{x_{n}\}$ and $\{y_{n}\}$ in *A* such that for all $\lambda \in [0,1]$

$$\begin{aligned} \lim _{n \rightarrow \infty }fx_{n} = \lim _{n \rightarrow \infty }S_{\lambda }x_{n}=\lim _{n \rightarrow \infty }gy_{n} = \lim _{n \rightarrow \infty }T_{\lambda }y_{n} = t \end{aligned}$$

where $$S_{\lambda }x = W(Sx,q,\lambda )$$ and $$T_{\lambda }y = W(Ty,q,\lambda ).$$

### **Definition 22**

Let (*X*, *d*) be a convex metric space and *A* and *B* be two nonempty subsets of *X* such that *B* is *q*-starshaped set. A pair (*f*, *S*) of two nonself-maps from *A* to *B* is said to be proximally commuting if for some $\lambda \in [0,1]$ whenever $d(x,W(Su,q,\lambda ))=d(y,fu)=d(A,B) \Longrightarrow W(Sy,q, \lambda ) = fx$.

If *A* and *B* are two nonempty subsets of a metric space (*X*, *d*), we define the following two sets.

$$\begin{aligned} A_0&= \{x\in A : d(x,y)=d(A,B) \quad {\text {for some}}\;y\in B\},\\ B_0&= \{y\in B : d(x,y)=d(A,B) \quad {\text {for some}}\; x\in A\}. \end{aligned}$$

### **Definition 23**

(Sankar Raj, preprint) If $A_{0} \ne \phi,$ then the pair (*A*, *B*) is said to have *P*-property if and only if for any $x_{1},x_{2} \in A_{0}$ and $y_{1},y_{2} \in B_{0}$

$$\begin{aligned} d(x_{1},y_{1})=d(A,B)\hbox { and }d(x_{2},y_{2}) = d(A,B) \Longrightarrow d(x_{1},x_{2})=d(y_{1},y_{2}). \end{aligned}$$

Now we presents a best proximity point theorem:

### **Theorem 24**

*Let* (*A*, *B*) *be a pair of nonempty, closed subsets of a convex metric space* (*X*, *d*). *Suppose that* *A* *is* *p*-*starshaped and* *B* *is* *q*-*stasrshaped set with Property (I)*. *Also suppose that* $A_{0}$ *is closed. Let* *f*, *g*, *S* *and*, *T* *be continuous nonself maps from* *A* *to* *B* *satisfying the conditions*:

(i) *Two pairs* (*f*, *S*) *and* (*g*, *T*) * satisfying common property* (*E*.*A*.) *w.r.t* *q* *and proximally  commuting*;
(ii) $$T(A) \subseteq f(A), S(A) \subseteq g(A), f(A_{0}) \subseteq B_{0}, g(A_{0}) \subseteq B_{0}$$;
(iii) *The pair* (*A*, *B*) *has* *P*-*property*;
(iv) *f*, *g*, *S* *and*, *T* *satisfying the condition* $d(Sx,Ty) \le \max \{d(fx,gy),dist(fx,[Sx,q]),dist(gy,[Ty,q]), \frac{1}{2}[dist(fx,[Ty,q])+d(gy,[Sx,q])]\};$
(v) *Two mappings* *S* *and* *T* *are* *pq*-*affine*.

*then* *f*, *g*, *S* *and* *T* *have a best proximity point.*

### *Proof*

For each $n \in N$, we define sequences $T_{n}: A \rightarrow B$ and $S_{n}: A \rightarrow B$ by $T_{n}y = W(Ty,q, \lambda _{n})$ and $S_{n}x =W(Sx,q, \lambda _{n})$ for all $x,y \in A$ and $\lambda _{n}$ is a sequence in (0, 1) such that $\lambda _{n} \rightarrow 1$

Consider

$$\begin{aligned} d(S_{n}x, T_{n}y) = d(W(Sx,q, \lambda _{n}), W(Ty,q, \lambda _{n})). \end{aligned}$$

By using Property *(I)* for the set *B*

$$\begin{aligned}&d(W(Sx,q, \lambda _{n}), W(Ty,q, \lambda _{n})) \le \lambda _{n}d(Sx,Ty)\\&\quad \le \lambda _{n}\max \{d(fx,gy), dist(fx,[Sx,q]),dist(gy,[Ty,q]), \frac{1}{2}[dist(fx,[Ty,q])+ dist(gy,[Sx,q])]\}\\&\quad \le \lambda _{n}\max \{d(fx,gy), dist(fx,S_{n}x),dist(gy,T_{n}y), \frac{1}{2}[dist(fx,T_{n}y)+ dist(gy,S_{n}x)]\} \end{aligned}$$

$\Longrightarrow d(S_{n}x, T_{n}y) \le \lambda _{n}\max \{d(fx,gy), dist(fx,S_{n}x),dist(gy,T_{n}y), \frac{1}{2}[dist(fx,T_{n}y)+ dist(gy,S_{n}x)]\}$ Now $T(A) \subseteq f(A)$ we can prove that $T_{n}(A) \subseteq f(A)$. For this purpose, consider

$$\begin{aligned} y & \in T_{n}(A)\\ y&= T_{n}x\quad {\text{for}} \;{\text{some}}\; x \in A\\ y&= W(Tx,q, \lambda _{n}). \end{aligned}$$

*T* is *pq*-affine and *A* is *p*-starshaped set

$$\begin{aligned}&y = T(W(x,p,\lambda _{n})) \subseteq T(A) \subseteq f(A)\\&\Longrightarrow y \in f(A)\\&\Longrightarrow T_{n}(A) \subseteq f(A). \end{aligned}$$

Similarly it can be proved that $S_{n}(A) \subseteq f(A)$. Now $T_{n}(A)\subseteq f(A)$ so for fixed $x_{0} \in A$, there exists an element $x_{1} \in A$ such that $T_{n}x_{0} = fx_{1}$ similarly a point $x_{2} \in A$ can be chosen such that $S_{n}x_{1} = gx_{2}$, continuing in process, we can obtain a sequence $\{x_{2n}\} \in A$ such that

20 $$\begin{aligned} fx_{2n+1} = T_{n}x_{2n}\,\,{\text{and}} \,\, gx_{2n+2} = S_{n}x_{2n+1}. \end{aligned}$$

Since $f(A_{0}) \subseteq B_{0}$ and $g(A_{0}) \subseteq B_{0}$, there exists $\{u_{n}\} \in A_{0}$ such that

21 $$\begin{aligned} d(u_{2n}, fx_{2n+1}) = d(A,B)\,\, {\text{and}} \,\, d(u_{2n+1}, gx_{2n+2}) = d(A,B). \end{aligned}$$

As the pair (*A*, *B*) has *P*-property then by Eq. (21) $d(u_{2n},u_{2n+1}) = d(fx_{2n+1},gx_{2n+2}).$

By using Eq. (20), we have $d(u_{2n},u_{2n+1}) = d(T_{n}x_{2n}, S_{n}x_{2n+1}). \text{Using Property (I) and the condition (iv), we can write the expression }$

$$\begin{aligned} d(S_{n}x_{2n+1},T_{n}x_{2n})& \le \lambda _{n}\max \{d(fx_{2n+_1},gx_{2n}),d(fx_{2n+1},S_{n}x_{2n+1}),d(gx_{2n}, T_{n}x_{2n}), \frac{1}{2}[d(fx_{2n+1}, T_{n}x_{2n})+d(gx_{2n},S_{n}x_{2n+1})]\}\\&= \lambda _{n}\max \{d(u_{2n},u_{2n-1}), d(u_{2n},u_{2n+1}),d(u_{2n-1},u_{2n}),\frac{1}{2}[d(u_{2n},u_{2n})+d(u_{2n-1},u_{2n+1})]\}\\&= \lambda _{n}\max \{d(u_{2n},u_{2n-1}), d(u_{2n},u_{2n+1}), \frac{1}{2}d(u_{2n-1},u_{2n+1})\}. \end{aligned}$$

$$\text{Hence} \hspace{0.5cm} d(u_{2n},u_{2n+1}) \le \lambda _{n}\max \{d(u_{2n},u_{2n-1}), d(u_{2n},u_{2n+1}), \frac{1}{2}d(u_{2n-1},u_{2n+1})\}$$

22 $$\begin{aligned} \Longrightarrow d(u_{2n},u_{2n+1}) \le \lambda _{n}d(u_{2n},u_{2n-1}). \end{aligned}$$

Similarly

23 $$\begin{aligned} d(u_{2n+1},u_{2n+2})&= d(fx_{2n+3},gx_{2n+2})=d(T_{n}x_{2n+2},S_{n}x_{2n+1})\nonumber \\ d(u_{2n+1},u_{2n+2})&= d(T_{n}x_{2n+2},S_{n}x_{2n+1})\hspace{0.5cm}\text{and}\nonumber \\ d(S_{n}x_{2n+1},T_{n}x_{2n+2}) & \le \lambda _{n}\max \{d(fx_{2n+1},gx_{2n+2}),d(fx_{2n+1},S_{n}x_{2n+1}),d(gx_{2n+2},T_{n}x_{2n+2}),\frac{1}{2}[d(fx_{2n+1},T_{n}x_{2n+2})+d(gx_{2n+2},S_{n}x_{2n+1})]\}\nonumber \\&= \lambda _{n}\max \{d(u_{2n},u_{2n+1}),d(u_{2n},u_{2n+1}),d(u_{2n+1},u_{2n+2}),\frac{1}{2}[d(u_{2n},u_{2n+2})+d(u_{2n+1},u_{2n+1})]\}\nonumber\\
\Longrightarrow(u_{2n+1},u_{2n+2}) & \le \,\lambda _{n}\max \{d(u_{2n},u_{2n+1}),d(u_{2n+1},u_{2n+2}),\frac{1}{2}d(u_{2n},u_{2n+2})\}\nonumber \\
\text{Hence}\hspace{0.5cm}(u_{2n+1},u_{2n+2}) & \le\, \lambda _{n}d(u_{2n},u_{2n+1}). \end{aligned}$$

From Eqs. (22) and (23), we obtain

24 $$\begin{aligned} d(u_{n},u_{n+1}) \le d(u_{n-1},u_{n}). \end{aligned}$$

This implies

25 $$\begin{aligned} d(u_{n},u_{n+1}) \le (\lambda _{n})^{n}d(u_{0},u_{1}). \end{aligned}$$

Let $m,n \in \mathbb {N}$ and $m < n$, we have

$$\begin{aligned} d(u_{m}, u_{n})\le &\, d(u_{m}, u_{m+1})+d(u_{m+1}, u_{n})\\\le &\, d(u_{m}, u_{m+1})+ d(u_{m+1}, u_{m+2}) + d(u_{m+2}, u_{n})\\\le &\, d(u_{m},u_{m+1}) + d(u_{m+1}, u_{m+2})+\cdots +d(u_{n-1}, u_{n}). \end{aligned}$$

By using Eq. (25), we come across

$$\begin{aligned} d(u_{m},u_{n}) & \le (\lambda _{n})^{m}d(u_{0}, u_{1})+(\lambda _{n})^{m+1}d(u_{0}, u_{1})+\cdots +(\lambda _{n})^{n-1}d(u_{0}, u_{1})\\ & \le (\lambda _{n})^{m}[d(u_{0},u_{1})+\lambda _{n}d(u_{0},u_{1})+\cdots +(\lambda _{n})^{n-m-1}d(u_{0},u_{1})]\\&= (\lambda _{n})^{m}[1+\lambda _{n}]+ (\lambda _{n})^{2}+\cdots +(\lambda _{n})^{n-m-1}]d(u_{0},u_{1})\\&= (\lambda _{n})^{m}\left[ \frac{1}{1-\lambda _{n}}\right] d(u_{0},u_{1}) \rightarrow 0 \,\, {\text{when}}\,\, m \rightarrow \infty. \end{aligned}$$

$\Longrightarrow d(u_{m}, u_{n}) \rightarrow 0$ when $m \rightarrow \infty$ this implies $\{u_{n}\}$ is a Cauchy sequence. Since $\{u_{n}\} \subset A_{0}$ and $A_{0}$ is closed subset of the complete metric space (*X*, *d*), we can find $u \in A_{0}$ such that $\lim _{n \rightarrow \infty }u_{n} = u$.

Since (*f*, *S*) and (*g*, *T*) have common property (*E*.*A*.) with respect to *q* so there exists a sequence $\{u_{m}\}$ in *A* such that

$$\begin{aligned}&\lim _{m \rightarrow \infty }fu_{m} = \lim _{m \rightarrow \infty }S_{\lambda }u_{m}=\lim _{m \rightarrow \infty }gu_{m} = \lim _{m \rightarrow \infty }T_{\lambda }u_{m} = t\\&\lim _{m \rightarrow \infty }T_{\lambda }u_{m} = \lim _{m \rightarrow \infty }W(Tu_m, q, \lambda )\\&{\text{and}} \;\lim _{m \rightarrow \infty }S_{\lambda }u_m = \lim _{m \rightarrow \infty }W(Su_m, q, \lambda ). \end{aligned}$$

Since $\lambda _{n} \in (0,1)$, we have

$$\begin{aligned} \lim _{m \rightarrow \infty }T_{n}u_{m}&= \lim _{m \rightarrow \infty }W(Tu_{m}, q, \lambda _{n})\\&= \lim _{m \rightarrow \infty }T_{\lambda _{n}}(u_{m})\\&= t \in B. \end{aligned}$$

Thus, we have

26 $$\begin{aligned} \lim _{n \rightarrow \infty }gu_{n} = \lim _{n \rightarrow \infty }T_{n}u_{m} = t \in B. \end{aligned}$$

Similarly

$$\begin{aligned} \lim _{m \rightarrow \infty }S_{n}u_{m}&= \lim _{m \rightarrow \infty }W(Su_{m}, q, \lambda _{n})\\&= \lim _{m \rightarrow \infty }S_{\lambda _{n}}u_{m}\\&= t \in B. \end{aligned}$$

and so

27 $$\begin{aligned} \lim _{m \rightarrow \infty }fu_{m} = \lim _{m \rightarrow \infty }S_{n}u_{m} = t \in B. \end{aligned}$$

Hence in light of Eqs. (26) and (27), we obtain

28 $$\begin{aligned} \lim _{m \rightarrow \infty }fu_{m} = \lim _{m \rightarrow \infty }S_{n}u_{m} = \lim _{m \rightarrow \infty }gu_{m} = \lim _{m \rightarrow \infty }T_{n}u_{m} = t \in B. \end{aligned}$$

Since *f*, *g*, *S* and, *T* are continuous so $f,g,S_{n}$ and $T_{n}$ are continuous and $u_{n} \rightarrow u$. Then from Eq. (28)

29 $$\begin{aligned} fu=S_{n}u=gu=T_{n}u. \end{aligned}$$

Since $f(A_{0}) \subseteq B_{0}$, there exists $x \in A_{0}$ such that

30 $$\begin{aligned} d(x,fu) = d(x,gu)= d(x,S_{n}u) =d(x, T_{n}u) = d(A,B) .\end{aligned}$$

As $(f,S_{n})$ and $(g,T_{n})$ proximally commuting, so

31 $$\begin{aligned} fx=gx=S_{n}x=T_{n}x. \end{aligned}$$

Taking limit $n \rightarrow \infty$ in Eqs. (29) and (31) we have

32 $$\begin{aligned} fu=Su=Tu=gu \quad {\text{and}} \quad fx=gx=Sx=Tx. \end{aligned}$$

Since $f(A_{0}) \subseteq B_{0}$, there exists $z \in A_{0}$ such that

$$\begin{aligned} d(z,fx) = d(z,gx) = d(z,S_{n}x) = d(z,T_{n}x)= d(A,B). \end{aligned}$$

Because the pair (*A*, *B*) has *P*-property so $d(x,z) = d(S_{n}u, T_{n}x)$

$$\begin{aligned} d(x, z)&= d(S_{n}u, T_{n}x)\\ & \le \lambda _{n} \max \{d(fu,gx), dist(fu,S_{n}u), dist(gx,T_{n}x),\frac{1}{2}[dist(fu,T_{n}x)+dist(gx, S_{n}u)]\}\\ & \le (\lambda _{n}) \{d(x,z)\}. \end{aligned}$$

This implies that $(1-\lambda _{n})d(x,z) \le 0$. So, $x=z$ and hence

33 $$\begin{aligned} d(A,B)= d(x,fx) = d(x,gx) = d(x,Sx) =d(x, Tx). \end{aligned}$$

Suppose that y is another best proximity point of the mappings *f*, *g*, *S* and *T* such that

34 $$\begin{aligned} d(A,B)= d(y,fy) = d(y,gy) = d(y,Sy) =d(y, Ty). \end{aligned}$$

Using Eqn. (29) and  *P*-property for the pair (*A*, *B*), we get that $x=y$. $\square$

## Conclusion

In this note, we defined the common property (E.A.) in the context of convex metric space that means here we assign the algebraic structure to the common property (E.A.) that is already exists in metric space. Due to this, we have been able to obtained a set of common fixed point theorems in which to ensure the existence of common fixed points the condition of range of one set is contained in other is not required. Thus, this newly introduced concept plays a great role in solving many kinds of physical sciences problems which can be recast in terms of common fixed point problems.
